# Does the Short-Lived First Human Milk Bank of Pakistan Hold Any Promise for the Future?

**DOI:** 10.34763/jmotherandchild.20242801.d-24-00035

**Published:** 2024-10-07

**Authors:** Amna Zaheer, Areeba Ahsan, Anum Akbar

**Affiliations:** Department of Internal Medicine, Liaquat National Hospital and Medical College, Karachi, Pakistan; Department of Internal Medicine, Foundation University School of Health Sciences, Islamabad, Pakistan; Department of Pediatrics, University of Nebraska Medical Centre, Omaha, Nebraska, USA

Dear Editor,

Considering Pakistan’s recent short-lived first human milk bank [1], we are discussing the implications of this significant development on nutrition education, neonatal health outcomes, and promises for the future, including challenges in successfully implementing this practice in one of the Islamic countries.

Human milk banking has seen varying levels of acceptance and implementation across Asia, South Asia, and the Middle East. Historically, countries like India and Iran have established human milk banks that have shown promising outcomes in improving infant health. For example, India has numerous human milk banks that have been instrumental in reducing neonatal morbidity and mortality by providing life-saving nutrition to infants who cannot be breastfed by their mothers [7]. In contrast, many other countries have faced cultural and religious challenges in establishing similar facilities [8].

The introduction of a human milk bank in Pakistan could significantly reduce neonatal morbidity and mortality, addressing the nutritional needs of vulnerable infants, particularly preterm and low birthweight (LBW) babies, who may benefit significantly from access to human milk. Breast milk is known for its immunological and nutritional benefits, which are crucial for the growth and development of newborns. Studies have shown that access to donor human milk can reduce the incidence of necrotizing enterocolitis, sepsis, and other infections in preterm infants [10]. According to a study, by providing a safe and reliable source of human milk, the milk bank can improve the health outcomes of countless infants, ultimately contributing to lower infant mortality rates in the country [3].

In Pakistan, an Islamic country, the establishment of a human milk bank was celebrated only for a while because it was suspended after a few days of its inauguration due to religious reasons [2]. This is similar to the fate of human milk banks in other Islamic countries (Figure 1). Islamic law addresses the concept of “milk kinship,” which impacts the social and familial relationships of children who consume milk from the same donor. Navigating these religious sensitivities carefully is essential to ensure the milk bank’s operations align with cultural and religious norms. Scholars and healthcare providers must work together to educate the public and provide religiously appropriate guidelines for the use of donor milk [4]. In 2023, the United States healthcare system made significant efforts to provide donor human breast milk to the Muslim community. These efforts led to Islamic scholars in Minnesota issuing a fatwa that encouraged the use of donor human breast milk [6].

**Figure 1.**
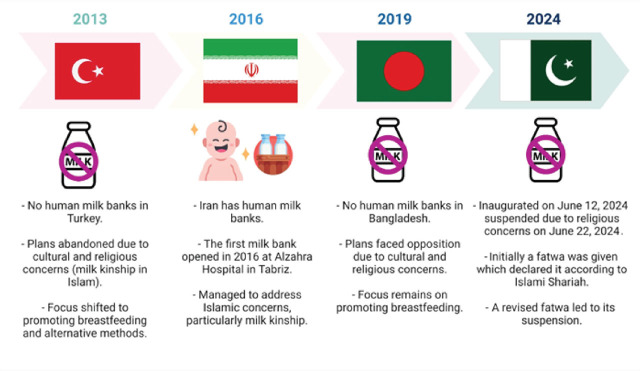
Human milk banks in other Islamic countries.

The successful implementation of a human milk bank in Pakistan will require addressing several challenges besides religious concerns, including establishing rigorous screening and pasteurization processes, ensuring equitable access to donor milk, and fostering collaborations between public and private healthcare sectors. Additionally, policies must be implemented to support and regulate milk donation and distribution effectively. The significance of educating and raising public knowledge about the advantages of human milk banking cannot be overstated. A comprehensive public education campaign should be launched to educate parents, caregivers, and healthcare professionals about the benefits of donor milk and the safety measures that must be taken to guarantee its quality. This campaign should also address common misconceptions and cultural barriers that may hinder the acceptance of milk banking [5,9].

## References

[1] Agencies (2024). Pakistan’s first Shariah-compliant human milk bank launched at SICHN.

[2] Bhatti M. W. (2024). Pakistan’s first Human Milk Bank ‘suspended’ over religious concerns.

[3] Committee on Nutrition, Section on Breastfeeding, Committee on Fetus and Newborn (2017). Donor human milk for the high-risk infant: Preparation, safety, and usage options in the United States. Pediatrics.

[4] Karadag A., Ozdemir R., Ak M., Ozer A., Dogan D. G., Elkiran O. (2015). Human milk banking and milk kinship: Perspectives of mothers in a Muslim country. Journal of Tropical Pediatrics.

[5] Mantri N., Goel A. D., Joshi N. K., Bhardwaj P., Gautam V., Gupta M. K. (2022). Challenges in implementation of mother milk banks in Rajasthan: A situational analysis. Journal of Mother and Child.

[6] Minnesota C. (2023). First-of-its-kind Islamic fatwa issued encouraging the use of pasteurized donor breast milk for Muslim babies in the hospital.

[7] Mondkar J., Chugh Sachdeva R., Shanbhag S. (2018). Understanding barriers and facilitators for human milk banking among service providers, mothers, and influencers of preterm and sick neonates admitted at two health facilities in a metropolitan city in India. Breastfeeding Medicine.

[8] Onat G., Karakoç H. (2019). Informal breast milk sharing in a Muslim country: The frequency, practice, risk perception, and risk reduction strategies used by mothers. Breastfeeding Medicine.

[9] Taksande A. A., Tote S., Taksande A., Javvaji C. K. (2024). Knowledge, attitudes, and perceptions of medical and paramedical students toward human milk banks and breast milk donation. Cureus.

[10] Tran H. T., Nguyen T. T., Mathisen R. (2020). The use of human donor milk. BMJ.

